# Statistical Reports on the Sickness, Mortality, and Invaliding among Her Majesty's Troops Serving in Ceylon, the Tenasserim Provinces, and the Burmese Empire

**Published:** 1843-01

**Authors:** 


### BRITISH AND FOREIGN
### MEDICAL REVIEW,

FOR JANUARY, 1843.

### PART FIRST.

### Analytical and Critical Reviews.

### Art. I.

. *Statistical Reports on the Sickness, Mortality, and Invaliding among Her Majesty's Troops serving in Ceylon, the Tenasserim Provinces, and the Burmese Empire. Prepared from the Records of the Army Medical Department and War-office Returns. Presented to both Houses of Parliament by command of Her Majesty.—London,* 1841.

2. *Statistical Reports on the Health of the Navy for the years* 1830, 1831, 1832, 1833, 1834, 1835, *and* 1836. *South American, West Indian, and North American, Mediterranean, and Peninsular Commands. Ordered by the House of Commons to be printed,* 1840.

3. *Statistical Reports on the Health of the Navy, for the years* 1830, 1831, 1832, 1833, 1834, 1835, *and* 1836. Part II. (*Cape of Good Hope and West Coast of Africa, and East India Commands, Home, and various forces.*) *Ordered by the House of Commons to be printed,* 1841.

The former Reports of Major Tulloch have received at our hands that full share of extract and comment which we are in the habit of bestowing on important and valuable works; and as the present volume supports to the fullest extent the character of its predecessors, an ample view of its contents shall be laid before our readers. We have associated with it the Naval Reports to which we always intended to revert; and the present we think a very suitable occasion for doing so, for reasons assigned in the following extract from the "Introduction" of Major Tulloch's Report:

"In connexion with these investigations a condensed series of reports on the health of the navy was last year presented to Parliament, which, besides affording a most useful means of comparing the relative health of seamen and soldiers, supplies excellent corroborative evidence of the accuracy of the deductions in this and the previous volumes, as to the remarkable immunity from endemic fevers at some tropical stations, where the alleged causes of it are obviously abundant; and also, in regard to the general prevalence of consumption and other affections of the lungs in the naval and military force employed in the Mediterranean—points on which, without such corroborative evidence, some doubts might perhaps have been entertained."

We concur with Major Tulloch as to the importance of the testimony derived from the Naval Reports, in corroboration of his own conclusions regarding the causes of disease. But we would remark, moreover, that no inconsiderable portion of the value of each succeeding Military Report depends on the opportunity of comparison on this head between it and its predecessors; for from no quarter can Major Tulloch's deductions derive more important or authentic confirmation than from his own further researches. At the very commencement of his Report we find a topographical and statistical view of Ceylon, having an important bearing on this point; and we shall extract from it those portions which have the most direct reference to a subject as interesting to us as to the gallant author.

The Island of Ceylon lies on the western side of the Bay of Bengal, between 5° 54′ and 9° 50′ north latitude, and 79° 50′ and 82° 10′ east longitude; and measures 270 miles in extreme length, 145 in extreme breadth, and includes an area of about 24,664 square miles. It is bounded by a wide expanse of ocean in every direction except on the north-west, where the gulf of Manar—about twenty miles in width—separates it from the continent of India.

The number of inhabitants is estimated at 1,100,000, or about 44 to the square mile. So limited a population in an island nearly equalling Ireland in extent, shows that the greater part must be uncultivated; in 1831, out of more than 2,000,000 of acres, only 300,000 were under crop, and 76,000 under pasturage.

Ceylon is naturally divided into two extensive tracts of country—the upper and lower; the former occupying nearly the centre of the island, and consisting of a broken mass of highlands, towering to the height of many thousand feet; while the lower is merely a level belt of ground, varying from thirty to forty miles in breadth. Except in the vicinity of the principal villages or lines of communication, this flat belt of land, which stretches in every direction to the sea-coast, is in a great measure covered with forests, and is frequently wet and marshy, owing to the streams from the high grounds overspreading the surface during heavy rains, and evaporation being retarded by the dense foliage, which excludes the solar rays; while universal gloom and stillness indicate the locality as unfavorable to animal life. In some parts, however, the low grounds present a different aspect; and as the sea-coast is approached, indications of life and industry become more frequent, and cultivation is found rapidly advancing under the exertions of an increasing population. Along the banks of many of the streams, particularly at their embouchure, numerous villages are to be met with; and in the districts of Galle and Colombo, which are the most populous, the inhabitants average from 150 to 160 per square mile.

The interior of the island is described as consisting of a succession of isolated hills, for the most part of a conical or oblong shape, but in a a few instances stretching into a table land of considerable extent, rising rapidly over each other from the level belt of land above described, to the height in some cases of above 8000 feet. The valleys of the interior are exceedingly fertile, and, owing to the facility of irrigation, are generally cultivated with rice; the hills are seldom brought under cultivation, though well adapted for every description of tropical produce. Except on the southern slope of the highlands, where the country is open, forest and underwood clothe the surface with the same dense covering as in the low grounds; and during heavy rains the rivulets rush down with such impetuosity as to inundate the level grounds along their course, and this abundant supply of moisture, aided by the power of a tropical sun, speedily gives birth to such a density of vegetation as to render the uncleared valleys nearly impassable.

Owing to its proximity to the equator, Ceylon may be said to possess a perpetual summer, varied only by the occasional difference of a few degrees of temperature, or by a change from wet to dry weather, or the reverse. These changes are principally effected by the south-west and north-east monsoons, the former of which extends from April or May to October, the latter from November to February or March. The south-west monsoon reaches the island cool, invigorating, and saturated with humidity, after having traversed a vast expanse of ocean. The north-east monsoon arrives at Ceylon under very different circumstances; for the more that wind inclines to the north, the more land it has to pass over before reaching the island. Owing to the modified influence of these winds in some parts compared with others, very considerable difference prevails in the relative degrees of moisture, and in the periods at which most rain falls. The moisture of the south-west monsoon is checked in its progress across the island by the highlands of the interior, and rapidly condensing, pours in torrents on the stations to the south and west; but the same effect is not experienced to the same extent from the north-east monsoon; both because the wind is less saturated with moisture, and because there are no hills in the district to aid its condensation, much of it passes over the coast and falls either on the highlands of the interior, or on the stations to the south-west. Thus, while in Trincomalee there is seldom any rain, except during the north-east monsoon, at Colombo and Galle showers are frequent, not only during the whole continuance of the south-west monsoon, but throughout the year; consequently the districts of the former have generally an arid and parched appearance compared with the latter.

There is no hygrometric measurement of the average dampness of Ceylon; but that it exists in a considerable degree in the lower grounds is indicated by the ready deliquescence of salt and sugar there, and the tendency of metals to corrosion. The fall of rain on the south-west coast averages from 70 to 80 inches, and on the north-east from 50 to 56 inches annually. Dew, however, is rarely deposited in any quantity, owing to the equality of the temperature of the day and night. Ceylon possesses a mean annual temperature of about 80°. It varies at the different stations occupied by our troops, according to their elevation. At Colombo, on the coast, the annual range of the thermometer is from 88½° to 74°; at Trincomalee, likewise on the coast, from about 91° to 75°; at Kandy, 1676 feet above the level of the sea, from 84° to 60°; at Niuera Elia from about 69° to 41°. Along the coast the daily variations of temperature rarely exceed 10° or 12°; but at Kandy a difference of 20° has sometimes been observed; and at Niuera Elia it has been seen to amount to 37°.

In Ceylon, then, the reputed causes of disease—the moisture, the high temperature, and the jungle—exist in abundance; nor does the medical history of our troops there belie its character. The admissions into hospitals are at the rate of 1678 per 1000 of the mean strength, and the deaths 69·8 per 1000, or, some allowances being made for omissions in the medical returns supplied from those of the War-office, 75 per 1000—a mortality nearly four times as high as in the United Kingdom, and only 6 per 1000 lower than that of the Windward and Leeward command in the West Indies. Of this very considerable mortality we find nearly 24·6 per 1000 arising from fever of different types, of which common continued is of the most frequent occurrence, and remittent the most fatal. Of 20,846 cases of fever admitted into hospital during the period of twenty years—from 1817 to 1836, inclusive—comprised in the Report, there were 10,965 cases of common continued fever, 5238 of intermittent, and 4643 of remittent. Of those admitted with common continued fever, one in 130 died; of those with intermittent, one in 50; and of those with remittent, one in 5½.

I. Fever. The continued fever of Ceylon is a tractable disease, and is not supposed to arise from any malarious influence; but rather from solar heat, intoxication, or other sources of excitement. The intermittents in the general return appear numerous, but when we learn that nearly two thirds of the cases took place in 1818 and 1819, when the troops were employed in very arduous service, and much exposed to the inclemency of the weather, we are led to the same conclusion as Major Tulloch, that "the proportion is very low indeed in an island having so moist a climate, covered with wood, and abounding in all the agencies which are supposed to induce fevers of this type." This view is confirmed when we remark that from 1825 to 1836 inclusive, the number admitted for this disease did not exceed 22 per 1000 of the strength annually, a tenth part of those occurring in the West Indies, with a *similar* climate, and a country better cleared and more under cultivation.

The remittent fever of Ceylon is a singularly deadly disease, as is manifest from it proving fatal to nearly one in five of those attacked, a virulence exceeding that of the yellow fever of the West Indies, which is fatal to one in eight; indeed about one third of the entire mortality among our troops during twenty years of service there has been owing to it. It is subject to epidemic aggravations, which have not been universal but confined to particular localities; and, it would appear, that although, as a general rule, the situations in which these aggravations have occurred have possessed an ample share of inundation and jungle, they have not done so in a very eminent degree, compared with the rest of the island. The most striking of several instances of this epidemic prevalence occurred in the district of Welasse in 1818. This district is described as extending from the eastern slope of the hills, which bound the elevated terrace of the interior to the sea-coast; and as being, from the base of the hills to the sea, low, flat, and subject to frequent inundations during the wet season, in most parts covered with forest and jungle, but in others possessing a considerable extent of rice cultivation. Kottabowa, however, the principal station in the district, and where the sickness prevailed, stands on rising ground overlooking the level tract, and its vicinity was tolerably well cleared and free from jungle. The total force in the district was 254; of these there perished of the fever, between the 12th of July and the 20th of October, 209; and of the 45 remaining on the 20th of October, the great majority are understood to have died before the end of the year. Unfortunately it was necessary to *march* a division of 192 men belonging to the 86th regiment through this most pestilential district. Though they all left the coast in excellent health, 125 were attacked by remittent fever, and 29 died between the 20th of October and 20th of December of that year; and at the latter date 30 were still in hospital, of whom several also died. In no part of the West Indies, nor even on the west coast of Africa, observes Major Tulloch, has fever ever assumed so severe and fatal a form. Whether, however, it would be the same in all years in that part of the country there are no means of determining, as white troops have not since been stationed there.

In other cases, however, it has been evident that an epidemic similar to the foregoing has formed a contrast with the ordinary sickness of the district. Such an epidemic appeared at 1824 at Kandy, which post we have already described as situated at an elevation of 1676 feet above the level of the sea; and as, consequently, enjoying a more moderate temperature than the flat country of the island. Eighty-two inches of rain fall, on the average, in the year; and the soil is described as consisting of siliceous and argillaceous particles, the detritus of the neighbouring mountains, mixed with small portions of calcareous matter. We find that the highest rate of annual mortality of white troops prior and subsequent to 1824 was 80 per 1000, the lowest was 12, and the average of 16 years, exclusive of the epidemic year, was 43 and a fraction, whereas in 1824 it amounted to the ratio of 333 per 1000, in other words to one third of the entire force. This prodigious mortality arose mainly from the prevalence and fatality of remittent, of which there perished at Kandy and Kornegalle, a place thirty miles to the north-west of Kandy, whence a small garrison was furnished to it, 113 of a total force amounting to about 350. During the epidemic there was likewise an increase of intermittents.

"A slight increase of temperature," remarks Major Tulloch, "and a longer continuance of dry weather than usual, were the only circumstances which preceded or marked the continuance of this epidemic; but its subsequent reappearance in 1824 and July 1825 was not marked by any such indications, and, since then, every variety of season, hot and cold, wet and dry, equable and changeable, has passed over without inducing a greater extent of febrile disease than would be likely to occur among an equal number of troops in the most healthy of our colonies." (Tulloch's Report, p. 30.)

Of all inscrutable matters connected with etiology, these epidemic aggravations of endemics are the most inscrutable. Their very range is matter of dispute; as, for instance, it is still disputed whether the yellow fever of America and the Spanish coast be or be not a mere aggravation of the remittent ordinarily existing there. In the case of Ceylon there is no dispute; we find no intimation of any suspicion of a new or unwonted disease introduced *ab extra*, as has often been supposed to be the case with yellow fever; the disease prevailing was too manifestly only a more abundant crop of the ordinary fruit of the climate. But when we endeavour to search the causes of such unwonted prevalence, how involved in darkness do we find them! A common supposition is that diseases not ordinarily contagious, as remittent fever for example, become invested with this property. But this is merely carrying the difficulty a step further back, for it meets us in the question, "whence this contagious quality?" which we are unable to answer.

We quit these statements of Major Tulloch regarding fever in Ceylon, to place in contrast with them details from a region which, though situated in an opposite hemisphere, abounds equally with Ceylon in the high temperature, the marsh, and the moisture, and what are considered "the appliances and means" of pestilence, without engendering it—South America. After describing the great extent of coast and cruising ground in the South American command, Dr. Wilson, in the Naval Report, proceeds to say:

"The places principally frequented are Rio de Janeiro, Buenos Ayres, Bahia, Pernambuco, and Para, Valparaiso, Callao, Coquimbo, Panama, and San Blas. In some of them art has been busy and effective; large towns have been built, and the ground has been cleared and cultivated in the neighbourhood of the towns, and on the margins of the bays and harbours; in others, beyond rearing houses and stores, things remain very nearly in their natural condition. Hence extensive marshes are in close contact with many of them; while in others, but more from natural formation than the labours of man, dry land, little productive of vegetable matter, abounds. The places named are, with three exceptions, situated within the tropics, some near their external limits, some close to the equator, and others at different intermediate points; yet with all such difference in position, soil, products of soil, and climatorial heat, the inhabitants of the shores of this vast continent, whether permanent or occasional, enjoy a high and singularly uniform degree of health.

"For the health enjoyed it is difficult, impossible, according to commonly received opinions, to account. Epidemic diseases are scarcely known; with wide-spreading and destructive force they are totally unknown. The fever which frequently makes such havoc in the West Indies never makes its appearance here. To such fevers as those which are so fatal in Asia and Africa, in North America, and even in the Mediterranean, the people of this continent are not subject. They are not free from febrile disease, but they suffer little from it, and from severe sweeping epidemics of all kinds they are exempt. What is the cause of such immunity? Why is it that in a land-locked harbour in this part of the world, under a powerful sun, surrounded by marshes and rank vegetation, ships lie for months or years without the occurrence of a single case of concentrated fever; while in other places, in Africa, in Asia, in North America, and more especially in the West Indian islands, things, to superficial observation, which appear to be the same are productive of so much disease and death?" (Naval Report, No. i. pp. 4-5.)

The copious returns in the Naval Report justify to the fullest extent these general statements of Dr. Wilson. In the year 1832, for example, the ratio of mortality was 6·2 per 1000 of mean strength, every instance of death, whether from disease or accident, being comprised. The Warspite, with an average complement of 600 men, lay the whole year in Rio Janeiro harbour, and did not lose a man, and had only seven cases of fever, viz., two of intermittent, four of gastric-remittent, and one catarrhal.

We now turn, for the sake of comparison, or rather contrast, to a region the insalubrity of a portion of which to the European constitution has long been well known to the British, viz. the West Indies and North America. From the Naval Reports we learn that in the squadron there employed the sickness had been moderate from 1831 to 1834, inclusive, but still with a progressive increase in the class of diseases—the febrile—on which mortality in that region is mainly dependent; each succeeding year being more unhealthy than its predecessor. We shall state the rate of progression in this class. In 1831 the mortality from fever was in the ratio of 4·8 per 1000 of the mean strength; in 1832 it was 5 per 1000; in 1833, 6·8 per 1000; and in 1834, 11·8 per 1000. When we reach 1835, however, there is a very sudden advance indeed in the general mortality, and especially in that from fever. In 1831 the ratio was 11·7 per 1000 of the mean strength; in 1832, 11·4 per 1000; in 1833, 16·3 per 1000; and in 1834, 19·8 per 1000; whilst in 1835, it was 37·5 per 1000. In the febrile class, in 1835 the total mortality was 98, being in the ratio of 30·6 per 1000 of the mean strength employed, and 1009 being the number attacked, is that of 97·1 per 1000 of those who sickened.

The succeeding year, 1836, presents a marked contrast to its predecessor. The total deaths from febrile diseases during it were 13, being in the ratio of 3·7 per 1000 of the mean strength; whilst the total deaths from all diseases were 32, being 9·2 per 1000 of the mean, or in the former case, one seventh of the mortality in 1835, in the latter, one fourth.

Discrepancies so striking as these between two consecutive years, and the gradual growth during five years of the endemic till, in 1835, it attained its maximum epidemic prevalence, to sink in 1836 into a dormant state to recruit its energies for a fresh career, are facts which cannot fail to set the mind on reflecting and speculating as to their causes. Without at all thinking it sufficient to account for the great increase of fever, we are of opinion that a circumstance not noticed by Dr. Wilson,—viz. the great increase of the naval force in the West Indies during these latter years, owing to the approaching emancipation of the slaves,—had considerable effect in swelling the list of the numbers attacked. Dr. Wilson's remarks are sensible and moderate. They may be charged with want of definiteness and clearness; but who can be clear on a subject consisting only of an assemblage of facts unreferred as yet to any general law—that which we had fondly framed for their explanation having proved fallacious?

"The mortality," says Dr. Wilson, "has been progressively increasing through a period of five years in the united squadron; the rate of increase being proportionate to, and dependent on increase in the force of febrile disease. Progressive increase of this sort in West Indian fever and other endemic diseases, during a number of consecutive years, tending to, and terminating in a general distribution of its agency, is often observed. It may be represented, in a general way, as taking the following course: After the lapse of a year or two, in which it can scarcely be traced, it occurs rarely or separately during another year or two. During another similar period it will arise more frequently, but still with considerable intervals of time and space; then, connected perhaps with a particular state of the weather, it bursts forth simultaneously at various points of the island and its harbours; the endemic becomes epidemic, attacking at once ship, regiments, and citizens, and carrying off great numbers. After such a course, though it may linger a little with greatly reduced force in some places, a period, longer or shorter, of exemption ensues. The cause of the disease seems exhausted for a time, the stock accumulated—so to speak—worn out, and a certain period is necessary to its reproduction.

"What is the meaning of this progress? What is the reason why an epidemic, having been extinguished, reappears, first in solitary and detached instances, then more frequently and in closer connexion, gathering strength as it proceeds, and finally explodes with the renewed power of a sweeping epidemic, again to be extinguished and again to be renewed? It would appear that the cause of the disease is associated intimately and necessarily with a peculiar local influence—a morbific atmosphere, emanating from, or not far from, the place where the disease is excited; that the local morbific atmosphere is occasioned by certain inappreciable actions carried on with more or less intensity at or near the surface of the earth; that these actions proceed at different rates at different times, and under modifying circumstances, their products being also different, not in kind, but in degree; and that the disease has close, continual relation to these actions and their morbific results, being rare at their commencement, more frequent as they proceed, and rife when they are complete. It is probable that degrees of heat and moisture, and other anterior agencies, as well as local circumstances, acting on individuals, influence the cause or the subject of the disease, or both, and that they have the power of precipitating or retarding, of lessening or augmenting the force of the essential cause." (Naval Report, No. i., pp. 108-9.)

The details of sickness in the Mediterranean and Peninsular squadron present, so far as fever is concerned, a very different picture from the foregoing. The proportion of cases and deaths, though not uniform, shows neither the steady movement from year to year observed during one period in the West Indies, nor the sudden exaltation of the disease into an epidemic which distinguished another. The loss was small, being under 2 per 1000 annually of those employed; greater, certainly, than in that very remarkable country, South America, where the average deaths amounted only to 1·3 per 1000, but small and insignificant when compared with that in the West Indies.

We shall continue the subject of fever through the Cape of Good Hope and the west coast of Africa commands. In the case of the navy, where ships are continually moving from one point to another, it is impossible to appropriate to each its due share of the causes of disease and death; the naval returns presenting, in this respect, a contrast with the precision which the Military Reports derive from stationary garrisons. The present Report, then, comprising the whole of an enormously extensive cruising ground, must be expected to differ widely in its results from the different portions of the Military Report which we have already passed in review, (Brit and For. Med. Rev., Vol. XI., p. 365,) representing neither the extreme salubrity of the Cape, nor the deadliness of that most pestilential of regions, the tropical part of the west coast of Africa. Of all forms of essential fever there were, in the seven years comprised in the Reports, 1587 cases, being in the ratio of 149·8 per 1000, annually of the force; of these 57 were invalided, and 135 were fatal; or in the ratio of 5·4 per 1000 of the former, and 12·7 of the latter. The destruction of life from all causes amounted, during the seven years, to 25 per 1000, on the annual average of the force. Notwithstanding that the west coast of Africa occupied little less than one half of the force, yet is the general mortality, and especially that from fever, lower than we should have expected. The lowness of the rate receives from Dr. Wilson the additional explanation, that fever, in a concentrated form, was not very prevalent during the period comprised in the Report: it happened that immunity to a considerable extent, compared with preceding and following years, was enjoyed during the period.

The Naval Report brings us now again to the point whence we started to trace the progress of fever round the world—the East Indian command. This extensive command embraces 70 degrees of latitude, and 100 of longitude, extending, in one direction, from the Tropic of Cancer to the 45th degree of south latitude; and in another, from the 50th to the 150th of east longitude. The northern limit is the isthmus of Suez, the southern the island of Tasmania. Notwithstanding the remote boundaries of the command, the operations of the squadron are principally directed to the shores of the bay of Bengal, of the coast of Coromandel, and of the *island of Ceylon.* From this statement it is manifest that the service of the squadron is, in a large proportion, intertropical, a great portion of it close to the equator. Yet, though the heat is necessarily high, and though the shores frequented abound with the reputed causes of violent disease, we find, on surveying these returns, the general mortality and that from fever much less than might be apprehended. The mortality from all causes was 17·3 per 1000, per annum, and that from fever 3 per 1000 per annum, of the force employed. There were 2302 cases of fever admitted during the seven years, of which but 39 were fatal from a total force of all classes, men and officers, of 12,948. Whereas, by reverting to Major Tulloch's Ceylon Report, we find that there the admissions from fever, out of a total force of 42,978, amounted to 20,846, or in the annual ratio of 485 per 1000 of mean strength, and the deaths from fever were 1056, being in the annual ratio of 24·6 per 1000 of mean strength. The total admissions from all diseases during the period were 72,100, being in the annual ratio of 1678 of mean strength; whilst the total deaths were 3000, or in the annual ratio of 69·8 per 1000. It thus appears that the total mortality was more than four times greater among soldiers than sailors, and the mortality from fever eight times greater; though, as we have already stated, the main cruising-ground of the squadron was intertropical, and a portion of it around the island where the sickness of the troops was comparatively so great. This appears the more remarkable when we consider that Trincomalee, where the greater part of the naval force is stationed, has a much more insalubrious character than any of the British settlements in the East, at least, so far as regards troops on shore; but it has always been remarked that the sickness which is so often prevalent among them very rarely extends to the shipping, though only a few hundred yards from the shore, with which the intercourse of the seamen is at all times free and unrestrained. We should not have expected that the difference of effect from occasionally touching on shore, and permanent residence there would have been so very great as it is thus shown to be. At all events, these comparative facts serve to settle the question, if there be any question regarding it, of the terrestrial origin of intertropical fevers.

To complete the sketch of the prevalence of fever in the navy we must pursue it through the branch of this service, described in the Reports as "the home and various force." By the "home force" is meant that employed in flag and other harbour duties; cruisers, employed in protecting the revenue, and by surveying vessels. There are three commands, —Portsmouth, Plymouth, and Sheerness. The force employed "variously," consists, if we understand right, of ships sent on special missions, such as from Spithead to Malta, the Cape of Good Hope, Bahia, and back again, and of ships fitting out. In the former case they are under circumstances highly conducive to health; in the latter, the crews having generally been collected recently, and from a state of indigence and profligacy, and, from a dread of their deserting, being at first ill supplied with body and bed clothes, one would naturally consider their circumstances as little favorable to health. The result in these forces so variously circumstanced, so far as fever is concerned, is thus stated by Dr. Wilson: The average proportion attacked with fever in the ships on the "home" stations, was 51·2 per 1000, while the deaths was 1 in 1188 of the mean number employed annually. In the "various" force, the ratio of attacks was little higher; being 60 per 1000; but that of deaths was nearly double, being 1 in 625 of the number employed annually. The following is Dr. Wilson's scale of severity of fever in the different regions of the world, as shown by the Naval Reports: first, the "home" force, in which the ratio of mortality was ·8; second, the South American, in which it was 1·3; third, the "various," in which it was 1·6; fourth, the Mediterranean, in which it was 1·8; fifth, the East Indian, in which it was 3; sixth, the West Indian and North American, in which it was 11·2; seventh, the African, in which it was 12·7 per 1000 annually of those employed.

In a general summary, written with much modesty and good sense, of the state of health of the entire navy, during the seven years comprehended in the Reports, we are informed that fever was the most fatal form of disease, more than a fourth part of all the mortality, including that from external causes, resulting from it; yet it was in the proportion only of 3·7 per 1000 annually of strength, the total number of deaths being 593, and the aggregate number employed 157,770, in the seven years. Dr. Wilson questions whether this statement of the really very moderate mortality, from essential or idiopathic fever is not beyond the reality; whether there may not be some confounding of symptomatic with primary or essential fever. From a careful perusal of these Reports we suspect Dr. Wilson's opinions on fever to be of a somewhat speculative nature; and we should deem it only right and prudent to consider the evidence of the medical officers immediately in charge of the sick as decisive of the nature of the disease they were treating. Indeed, to throw a doubt upon it, is to depreciate the whole Reports; for it is impossible to make the requisite correction for the supposed error. And, moreover, acquiescence in the conclusions as to the nature of the disease, come to by those in immediate attendance on the sick, if in any case right, is particularly so in the present; as we are convinced that a more intelligent or better-informed class of practitioners, whether in public or private life, is nowhere to be found than the medical officers of the navy.

Though not connected exclusively with fever, we find the progressive improvement of the health of the navy, so honorable to the discipline and interior economy, and, it may be too, to the skill of the medical officers of this right arm of England, that we take this opportunity of noticing Dr. Wilson's valuable information regarding it. For the period comprised in the Reports, the total deaths from disease in our navy, diffused as it is throughout every region of the globe, amounted to 11·8 per thousand of the force annually; and the total from all causes, disease and external injuries of every description, including deaths from drowning, the upsetting of boats, &c., to 13·8 per 1000; in other terms, one in 85 died annually from disease, and one in 72½ from all causes. To increase our exultation from this gratifying view, we learn that 30 years ago the mortality amounted to 31, and 60 years ago to 125 per 1000 annually of the force employed.

Sir Gilbert Blane, in a work published in 1822, stated that the proportion of deaths in the navy should not be more than 1 in 80 annually of the employed, supposing that the agents affecting life were not more detrimental there than among the civil population at corresponding ages. This estimate was formed on the erroneous supposition that the range of ages in the navy was from 20 to 40 years; it is shown in the Reports to be from 15 to 50, and yet Sir Gilbert's *desideratum* is more than realized within 20 years. What might not the result have been had his estimate of ages been correct!

The causes of these wonderful improvements are briefly stated by Dr. Wilson in the following terms:

"They consist in an almost entire change in the constitution of the service, especially in the following important particulars: abundance of nutritious food, and of wholesome palatable water; personal and general cleanliness, and comfortable clothing; ventilation, reduced allowance of spirits,* and afternoon meal of tea; small monthly payment of money, partly in addition to wages, since the year 1825; regulations and restrictions respecting punishments; provision for mental improvement and recreation; better-built ships, with greater capacity between decks; and, it may be added, without reflecting generally on the spirit and practice of former times, at least in so far as the executive was concerned, more judicious and humane treatment generally of seamen." (Report, Part ii, pp. 214-15.)

Two other not unimportant items in this most gratifying category of improvements, may with great propriety be here mentioned by us, although the official position in which Dr. Wilson stood, his modesty, and the public nature of his report, prevented him from noticing them—we mean, the very superior administration of the medical department of the navy by its present chief, Sir William Burnett,† and the greatly-increased knowledge and skill of the medical officers in charge of ships.

II. Diseases of the Lungs,—Phthisis. We shall now proceed to trace affections of the lungs in general, and consumption, particularly, through the extensive and diversified regions comprehended in the Reports.

In Ceylon, the numbers among the troops attacked with all diseases of the lungs were, on the average, 70 per 1000 annually, while the deaths were 4·1 per thousand of the mean strength of the troops. But the deaths from consumption alone amounted to 2·7 for 1000, leaving but 1·4 per thousand of mortality from inflammation of the lungs, acute and chronic catarrh, and asthma. The numbers attacked, too, with consumption, it should be remarked, amounted to 4·7 per 1000, being, in this singularly-equable climate, not one half lower than in the incle-

* We think this still much too large.

† Among the many improvements introduced by Sir W. Burnett into the medical department of the navy, we must particularly notice that of the "Sick Mess," (a greatly improved scale of diet for the sick,) for which the service is entirely indebted to him.

ment region of Canada, and in the inconstant climate of Great Britain, where the numbers attacked are 6·5 per 1000 annually. It should be observed, moreover, that all the cases of catarrh which terminated fatally in Ceylon (20 in number, or about 5 per 1000 of the mean strength) exhibited marks of tubercular disease, though not included under the head of consumption.

In the Tenasserim provinces, the total report of diseases of the lungs is in strict accordance with that from Ceylon as to the number attacked; but in strong contrast with it as to the proportion of consumptive cases, and consequently as to the total mortality. These provinces, formerly part of the Burmese empire, were ceded to the East India Company in 1826. They are situated on the eastern shores of the Bay of Bengal, and may be said to extend from lat. 10° to lat. 17° N., and from long. 97½° to long. 99° E., forming a narrow strip of territory on the continent of India, about 420 miles in length, and from 50 to 70 in breadth. The number attacked in this district, with diseases of the lungs, was the same as in Ceylon, 70 per 1000 of the strength annually. But the deaths, so far from being in the same ratio as in that colony, amounted to but 2 per 1000 annually; whereas in Ceylon they were 4·1 per 1000. This difference arises chiefly from the rarity of consumption in these provinces, but 4 cases having occurred during the period (10 years) of their occupation by our troops, or 1 to 1704 of the mean strength; of these four cases 3 were fatal. Hemoptysis observed nearly the same relative proportion as consumption in Ceylon and the Tenesserim provinces. The two diseases conjointly were in the latter situation in the proportion of 1 to 8 compared with Ceylon, and as 1 to 10 in comparison with the prevalence in Great Britain, or the Mediterranean. Major Tulloch acknowledges that the period of observation was too short, and the number of troops too small to warrant the conclusion that every year the proportion would be equally low; but he adds, that "this comparative exemption from consumption and hemoptysis extends, as will hereafter be shown, to most, if not to all, the stations throughout India, and constitutes a new feature in the history of these diseases, which will be more fully developed in a future Report." (Tulloch, Tenasserim Report, p. 9.)

Major Tulloch has a valuable report on the sickness and mortality of the troops employed in the Burmese war. The period, however, of the war was too brief, and the circumstances in which the troops were placed too peculiar to render it supposable that a fair inference could be deduced from our experience in the territory as to the influence of the climate on the European constitution. We shall therefore leave, as we did when fever was examined, this Report untouched so far as affections of the lungs are concerned, excepting that we concur in the opinion expressed by Major Tulloch, that the deaths from consumption having been but 2½ per 1000, this country in all probability partakes of the comparative immunity from this disease enjoyed by the Tenesserim provinces, and the whole of India.

From what has been already stated of the extreme salubrity of South America, a moderate return under the head of consumption might be expected from this quarter; but our expectations in this direction are more than fulfilled by the Naval Report. Of a strength consisting of 17,254, there were treated 55 cases of consumption, of which there were 25 fatal; the annual rate of mortality from the disease was, therefore, 15 per 1000 of the employed. From the circumstance that 30 out of the 55 were cured, Dr. Wilson deduced the very reasonable conclusion, that all the cases classed under the head of consumption, had not really belonged to the disease, a proportion having simulated it only : of 488 cases of inflammation of the lungs and their membranes, six only terminated fatally; and of 47 cases of hemoptysis, 4 terminated fatally ; so that from pneumonia, pleuritis, hemoptysis, and consumption, the total mortality was but 35, or little more than 2 per 1000 ; yet were affections of the lungs the most fatal diseases in this singularly healthy region.

In the West Indian and North American squadron, there were 519 cases of inflammation of the lungs and their membranes, of which 26 were invalided, and 22 fatal, the ratio of attacks being 22, of invalided ·11, and of dead ·9 per 1000 of mean force. Of consumptive cases 114 were treated, of which 44 were fatal, the attacks having been 4·8 per 1000, and the deaths 1·9. There is supposed to have been an error in the designation of the disease in some of these cases, 70 treated for consumption having recovered. We think, as in the case of South America, there is ample ground for the supposition.

We are strongly impressed with the smallness of the mortality of the navy, compared with the army ; but by far the most striking feature in the case is the discrepancy in the deaths from consumption in all climates, between these two branches of the public service. In the navy we see that in the West Indies and North America the mortality from consumption is at the rate of 1·9 per 1000. Now when we turn to the prevalence of consumption and the mortality from it among soldiers in the same region, we find such returns as the following: In the Windward and Leeward command 12 per 1000 are attacked annually with consumption ; in Barbadoes 15·8 per 1000 die of it; in Jamaica, where the disease is less frequent and less fatal than in other parts of the West Indies, the mortality from it is 7·5 per 1000. When we examine the returns from the more northern portions of the same command we find that in Nova Scotia and New Brunswick the deaths from diseases of the lungs among the troops are 7·1 per 1000 annually; in Canada the deaths from the same cause are 6·7 per 1000; and in Bermuda there are attacked with consumption annually 8·8 per 1000.*

When we travel homeward for a comparison we find soldiers dying of diseases of the lungs in an infinitely higher ratio than sailors in the West Indies and North America. Of the dragoon-guards and dragoons there die annually in this country from diseases of the lungs 7·7 per 1000; of the household cavalry, 8·1; of the soldiers of the West Indian depôts, 9·6; of the foot-guards, 14·1. When we look at the mortality from diseases of the lungs among our troops stationed in climates conspicuous for their exemption from such diseases, still do we find them more prevalent than among sailors afloat on the coasts of countries a portion of which—the West Indies—Major Tulloch has shown to be singularly favorable to the development of consumption, and in another part of

* Tulloch's Statistical Reports on Sickness, &c. in the West Indies, ditto in United Kingdom, Mediterranean, and British America; and Br. and For. Med. Rev., vol. VIII. p. 220 et seq.

which—Canada—the disease has been proved by the same high authority to be nearly as prevalent as in the United Kingdom. To select instances: In St. Helena the deaths from diseases of the lungs are 3·4, and at the Cape of Good Hope 3·9 per 1000 annually among the troops; whereas, among sailors in the West Indian and North American command, the deaths from consumption amount, as we have already shown, to but 1·9 per 1000; and those from all diseases of the lungs to less than 3·2 per 1000.

In whatsoever direction we try the comparison, we invariably find diseases of the lungs, and especially consumption, much less frequent and fatal among sailors than among soldiers. On this subject several reflections naturally suggest themselves. In the first place, how is the difference to be accounted for? Is it merely that the sailor is withdrawn from an atmosphere, it may be, close and confined, or abounding with terrestrial impregnations, or the smoke and corruption of the haunts of man into one of comparative purity and great freedom of ventilation; or is there some special preservative or even curative virtue in sea-air, derived, possibly, from its impregnation with chlorine, iodine, bromine, &c.? We send the phthisically disposed, or those already affected with phthisis, to climates in which consumption is quite as frequent as in England, in some cases even more so, yet are they benefited by it. May not the benefit arise from the voyage? and should we not better consult the interests of our patients by advising a course of voyages, than a residence in Malta, Madeira, or any other given point? It will be known to our literary readers that this very remedy was recommended many years ago by Dr. Gilchrist, in his treatise "On the Use of Sea Voyages in Medicine, Lond. 1756," and many cases given of its apparent efficacy. The same practice was earnestly advocated subsequently, in 1782, by Dr. Thomas Reid, in his "Essay on the Nature and Cure of Phthisis Pulmonalis." Both these authors attributed a principal share of the efficacy of the practice to sea-sickness, although Dr. Gilchrist, in particular, regards the saline atmosphere of the ocean-climate as also very beneficial. If we choose to go still higher for authorities, we find Aëtius lauding the excitement and inconveniences of a sea voyage as curative of obstinate chronic affections; and Celsus advocates the measure very strenuously, in the very disease now under consideration: "Opus est," he says, "si vires patiuntur, *longâ navigatione*, cœli mutatione, sic ut densius quam id est, ex quo discedit æger, petatur: ideoque aptissimé Alexandriam ex Italiâ itur." Now we are not hazarding too much when we conjecture that no steamer plied from Ostia to Alexandria in the days of Celsus, and that the consequent length of the voyage endowed Alexandria with much of its curative character. Indeed, that sailing is the main remedy of Celsus is indicated not merely by the phrase, "longâ navigatione," in the sentence quoted, but by his saying shortly afterwards, "Si id imbecillitas non sinit, *nave* tamen non longe, vectari commodissimum est." (De Re Medicâ, lib. iii., cap. 22.)

The opinions of Dr. Wilson accord with those of the high authorities we have quoted, and with our own, regarding the sanatory influence of sailing on the consumptively disposed and even the consumptive, as will be seen in his remarks made in a summary of the state of health of the navy in all regions, given in the Report of the "Home and Various Force." He in a subsequent passage, however, qualifies his opinion very considerably, and on grounds special to the service with which he is connected. Had he extended his views into a comparison between his own and the sister service or civil life, we think his original opinion would have received less qualification. His opinion is thus expressed:

"These Reports establish the important fact that sea life has sanatory influence on non-inflammatory affections of the lungs, at least on the most destructive one of them—the phthisical. The influence is chiefly preventive, but would appear to be also, in no small degree, curative. The preventive power shown by the comparatively low proportion of attacks, the curative by the large proportion of the attacked which appear by the returns to have been cured. The existence of a general sanatory influence is deduced, not only from the comparatively low proportion dying of the employed throughout the service, but also from the inconsiderable difference in the proportion dying in different commands, leading to the conclusion, that the diffused beneficial agency countervails the varying prejudicial power of parts and places resorted to. Whether there may be anything salutary in the victualling, apart from its nutritive effects is uncertain: it is certain there is nothing in their duties which, inasmuch as they conduce strongly to inflammatory disease of the pulmonary tissues, tend to excite, not prevent consumption." (Report of the Home and Various Force, pp. 207.)*

On this last point—the tendency of inflammatory disease of the pulmonic tissues to induce consumption—M. Louis and others might enter into controversy with our author. We, however do not purpose doing so, feeling more disposed to question the fact, whether the sailor's duties conduce so strongly to inflammatory disease of the lungs as Dr. Wilson, from this sentence, would seem to imagine. Certainly, looking at Dr.

* So favorable to the view of the great therapeutic influence of sea voyages in consumptive diseases are the results brought out in these admirable reports of Major Tulloch and Dr. Wilson, that it would seem no idle or foolish proposal to have a ship fitted out for the express purpose of taking consumptive invalids to sea and keeping them there for months or even years. It would be an easy matter to prepare and fit up in the most comfortable manner vessels for such a service; and, alas, it would be too easy to people many such from the crowds of victims of this fearful malady constantly found in that class of society whose members could *afford* to avail themselves of their advantages. We can imagine no better "Residence for the Consumptive," and no superior medical advantages than would be afforded by such a "Medical Yacht," commanded by an experienced naval captain, with Dr. Wilson or some other well-informed member of the medical staff of the navy as "viceroy over him," directing the course and progress of the ship, according to hygiènic and therapeutic principles. The Mediterranean sea, the better parts of the Atlantic and even of the Pacific ocean might thus be traversed—the inhabitants of the sea-city, like Ariel, still continuing "to fly after summer merrily."

Like benefits might be obtained, or, to speak more modestly, a similar experiment might be tried, on a smaller scale, and at a more moderate expense, by taking advantage of the innumerable ships which, as passage-vessels, or for purposes of commerce and colonization, leave our shores at all times and seasons, and for all parts of the world. An uninterrupted series of voyages might thus be taken in the steam packets to Malta or the West Indies and back; or in sailing vessels to the Brazils, India, China, Australia, the Sandwich Islands, Peru, or across the Pacific, returning by China. In this manner a residence at sea, of such length as might be desired, and almost such a climate as was thought most proper, could be obtained; and amid the multitude of ships it would not be difficult to select a good climate as a *land-residence* during the period of the vessel's detention at any port until her return-voyage. For instance, what would seem better than a long voyage to the Brazils, to Peru, to Australia, with a residence of a month or two on the shores or cool hills of these most salubrious countries, while the ship was preparing for her home-voyage?

Wilson's own returns of the fatality of inflammation of the lungs and their membranes in various commands, we are led to feel a doubt on the subject. From such diseases the "Home" and "Various" suffered more than any other division of the force, and exactly in the same proportion, the rate of mortality being 1·5 per 1000, annually, in each. The next highest is the Mediterranean, which is 1; the next is the West Indian and North American, which is ·9; the next the African, which is ·8; the next the South American, which is ·4; the lowest the East Indian, which is ·3 per 1000 of the employed.

A circumstance bearing on this point, and very interesting on many other accounts, is the remarkable fact that, while the proportion of cases of *consumption* is so low, that of *catarrh* is so high in the navy, being twice as high as in the army. The difference may probably be explained by greater exposure of the seamen to the inclemencies of the weather, especially during the night.

III. DISEASES OF THE LIVER. Passing from the subject of pulmonic to that of hepatic disease, Major Tulloch informs us that in Ceylon the admissions were in the ratio of 55 per 1000 annually; and the deaths in that of 4·9 per 1000, being very much higher indeed than in any station in the Western Hemisphere, Grenada in the West Indies alone excepted, where it amounts to 4·5 per 1000, only a small fraction less than in Ceylon. In other tropical climates in the Western Hemisphere, we find such returns of mortality from liver-disease in the army as the following: in the Windward and Leeward command (West Indies) 1·8 per 1000; in Jamaica, ·1; in Trinidad, 1·1; and, to turn to a temperate climate, 1 to10,000 in the foot-guards at home.* In the navy, the returns from the East Indies is 1·5 per 1000—very low indeed in comparison of the army at Ceylon; the "Home," ·5; the South American and African, ·4 each; the "Various," ·3; and the Mediterranean and West Indian, united with the North American, ·2 per 1000 annually.

IV. BOWEL COMPLAINTS. Before closing this article we shall give an abstract of the relative mortality in the army and navy, (so far as these Reports enable us to give it,) from a class of diseases, to which a great proportion of the deaths in the former force is always attributable, especially in tropical climates, those of the stomach and bowels. In Ceylon the deaths from these diseases amounted to 24·2 per 1000, of which mortality 23 per 1000 was owing to dysentery, a very high proportion, certainly, and exceeding that in the army, in the Windward and Leeward command (West Indies), where the deaths from the same disease are 15 per 1000. In respect of the prevalence of the disease, however, these stations in opposite hemispheres are nearly on equality, the difference in mortality arising from the greater severity of dysentery in Ceylon. In the navy the returns are as follows: in the South American squadron, from inflammatory affections of the bowels and dysentery, 1·6, and the West Indian and North American, 7 per 1000 annually. What a contrast does this last item present to the mortality from such diseases among soldiers in the West Indies, where, in the Windward and Leeward command, 20·7 per 1000 die annually of such complaints! In the Cape of Good Hope and west coast of Africa squadron, the deaths from dysen-

* Tulloch's West Indian Report, and Br. and For. Med. Rev., vol. IX. p. 64.

tery alone were 3·4 per 1000, whilst those from original inflammation of the stomach and bowels were very insignificant; the ratio of deaths being 1 in 2000 of the force. In the East Indian command the deaths from dysentery were 4·2 per 1000; those from cholera 2·4; and the collective deaths, from acute diseases of the alimentary canal constituted nearly one half of the total mortality from disease during seven years. But it should be remarked that this total mortality amounted only to 17·3 per 1000, or little more than two thirds of the deaths among soldiers from one disease, dysentery, in Ceylon, situated in the same hemisphere, and the same climate. What a view do these facts present to us of the relative salubrity of the pursuits of the soldier and the sailor! Is the occupation of the sailor especially salubrious, or must we conclude with Major Tulloch, that the military profession operates prejudicially to the health of those employed in it?

Throughout our investigations into the four great classes of diseases, whence the main mortality of our fleets and armies arises, Fevers, Pulmonary affections, Liver disease, and Inflammatory disorders of the Bowels; we have shown that an advantage exists in the former service infinitely greater than any preconceived opinion would have led to the belief in; and one which, as it appears to us, it is impossible to explain on the mere supposition that the sea service is especially salubrious. We are driven to the conclusion that there must be, besides, some peculiar circumstances which render the military service unpropitious to health, an opinion which the accurate investigations of Major Tulloch had led him to form a comparison between the mortality in the dragoon-guards and dragoons in the United Kingdom, and that of the civil population at the same period of life.* The exposure of our sailors to the terrestrial emanations on which endemic diseases are supposed to depend, being only occasional, would lead one to expect that they should possess an advantage in certain classes of diseases, fever, for example, and bowel complaints; but neither that it should be in any case so very decisive as it has been shown to be, nor that it should pervade every form of disease, of which we have traced the relative prevalence in the two services. Why, for example, do the two services in the same climate fare so differently from consumption and disease of the liver?—a question we are unable to answer, however we may have indulged in conjectures. We would suggest a diligent inquiry, in every climate, into the barracks, their site, ventilation, and space; the food, clothing, and duties of the troops, and above all, that general moral management applied to soldiers, from which we quoted Dr. Wilson's Report to prove such great benefit had resulted in the navy. On a former occasion we pointed out the fact how much bowel complaints in certain of our colonies had been diminished by the substitution of fresh for salt rations, (Brit. and For. Medical Review, vol. XI, pp. 67-8,) an amendment attributable, we believe, to Lord Howick. Might not some equally intelligent and patriotic secretary-at-war gain golden opinions by pursuing the path his lordship has indicated, and still further ameliorating the condition and health of our gallant soldiers? In this, and all similar investigations, our ministers would find an invaluable pioneer and counsellor in Major Tulloch.

* See Major Tulloch's First Report, and Br. and For. Med. Rev., vol. VIII. p. 224.

We have now brought to a close our observations on these Reports. So often have we testified to the merits of Major Tulloch, as a statist and even as a medical reasoner, that in his case it is only necessary to say that his present Report is on the plan of those which preceded it, and equals them in execution. In our previous article on the Naval Reports (Brit. and For. Med. Review, vol. XI. p. 182) we bore testimony to the editorial skill of Dr. Wilson, and his colleague Mr. George Mackeson, whilst we regretted that the defective system of nosology employed in the navy, (the best, however, at the period of its adoption,) impaired in no inconsiderable degree the value of their labours. Though we still think that the employment of an improved nosology would at once have diminished the amount of Dr. Wilson's labours, and increased the accuracy of their results, we cheerfully bear testimony to the very great value and extent of the information contained in his Reports, regarding it as a vast addition to the knowledge previously possessed in that important branch of our art,—etiology. We are of opinion, moreover, that the details given of the sickness, or rather of the health of the navy, are a proud eulogium on the hygienic arrangements of this branch of the service, and of the therapeutic skill of its medical staff.

P.S. Since the preceding pages went to the press we have seen, for the first time, a most valuable paper, by Major Tulloch, on the relative health of the Army and Navy, published in the Statistical Journal for Feb. 1841. We regret much that we had not the advantage of using this communication before; but we feel it necessary to lay before our readers several observations contained in it, also some derived from another source, as bearing most materially on the important discussion in the preceding pages.

I. The remarkable difference in the mortality of the two services may partly be attributable to the facilities in the navy for sending home patients whose recovery would be doubtful or protracted if they continued to serve abroad. The proportion *invalided* on this account in the navy, compared with the army, is as 25 to 10; consequently the former service gets rid of the greater proportion of the chronic cases, which, in the latter, continue in hospital till they terminate fatally.

II. In the navy there are frequent opportunities of sending home invalids as soon as that change is recommended, but in the army they could not, till within the last year or two, be sent home oftener than once or twice in twelve months, however urgent the necessity; consequently, numbers perished while waiting for a conveyance, whose lives would probably have been saved had the means of transport to their native country been afforded at an earlier period.

III. When invalids from the navy return home they are forthwith discharged, unless unable to leave the hospital; but in the army most of them who have not completed a sufficient period of service to entitle them to a discharge with pension, are ultimately sent back to their duty, and their deteriorated constitutions contribute materially to increase the mortality in after years.

IV. The comparatively short duration of the sailor's service has also a powerful influence in keeping the mortality below what prevails among military troops in the same climate. Soldiers are enlisted for life, and in most instances continue to serve from 21 to 25 years consecutively, while the average period of a sailor's engagement is only from three to four years; and as this engagement is, in every instance, preceded by a medical examination, at which all slight and sickly men are rejected, it must have a material influence in reducing the amount of mortality. The experience of Insurance offices shows the advantages of recent examination to be so great that several newly-established offices, consisting of 200 or 300 members have passed two or three years without a single death, a circumstance which never occurs among an equal number who have been longer assured.

v. Age also forms an important element in comparing the relative mortality of these two branches of the service. On this head the naval returns supply no detailed information, but it seems by no means probable that among men who only engage for a limited period the numbers at an advanced age can be as great as in the army, where the engagement is for life. In the navy, also, a large proportion consists of boys about the age of puberty, at which period of life persons are well known to be less subject to mortality than at any other.

This explanation will tend to relieve the results in the naval report from any suspicion of bordering on the marvellous, a character which might otherwise be assigned to them when the ratio of deaths from sickness in the Mediterranean is found to be under ten, and in South America under eight per thousand of the force annually, while that of the labouring population of this kingdom, at a corresponding period of life, is at least one half more. Even making all due allowance, however, for the probable operations of these causes in reducing the mortality, there is abundant reason for congratulation in the results, both to the philanthropist and the medical inquirer.

---

### Art. II.

*Erinnerungen an England.* 1841. Von Dr. Marx, Königl. Hannoverschem Hofrathe und Professor.—*Braunschweig*, 1842. 8vo, pp. 302.

*Recollections of England.* 1841. By Dr. K. F. H. Marx.

